# Falciform Ligament Appendagitis: A Rare Cause of Acute Epigastric Pain

**DOI:** 10.7759/cureus.99804

**Published:** 2025-12-21

**Authors:** Ahmed A Alali, Hamza M Ahmed

**Affiliations:** 1 General Practice, Al Nabaa Medical Center, A'ali, BHR; 2 General Surgery, Salmaniya Medical Complex, Manama, BHR

**Keywords:** abdominal pain, appendagitis, case report, falciform ligament, radiological diagnosis

## Abstract

Falciform ligament appendagitis is an exceedingly rare cause of acute abdominal pain resulting from inflammation and infarction of the falciform ligament’s fatty appendage. Its presentation often mimics more common causes of acute abdomen, making diagnosis challenging. We report the case of a 36-year-old Bangladeshi male with no prior medical history who presented with a four-day history of severe epigastric pain radiating to the right upper quadrant (RUQ). Clinical examination revealed localized tenderness and a palpable soft, well-circumscribed swelling, with laboratory investigations within normal limits except for mild leukocytosis and a slightly elevated alanine aminotransferase (ALT). Initial ultrasound suggested a rectus sheath hematoma; however, contrast-enhanced CT imaging demonstrated a fat-density lesion adjacent to the falciform ligament with surrounding inflammatory stranding, consistent with falciform ligament appendagitis. The patient was managed conservatively with analgesia, intravenous fluids, and supportive care, resulting in progressive improvement and complete resolution of symptoms by hospital day 4 without surgical intervention.

## Introduction

The falciform ligament is a fold of peritoneum that separates the liver’s left and right lobes and connects to the anterior abdominal wall. It contains the ligamentum teres hepatis, the remnant of the umbilical vein, and adjacent extraperitoneal fat [1]. Falciform ligament appendagitis is a very rare condition involving inflammation and infarction of the fatty tissue adjacent to the falciform ligament, often presenting with severe abdominal pain that can mimic more common causes of acute abdomen [2]. Here, we report a 36-year-old man who presented with severe right upper quadrant (RUQ) abdominal pain, later confirmed by contrast-enhanced CT imaging as falciform ligament appendagitis.

## Case presentation

A 36-year-old Bangladeshi male with no significant past medical history and no known drug allergies presented to the emergency department in July 2025 with a four-day history of severe epigastric pain radiating to the RUQ. The pain was constant and non-colicky, with no associated nausea, vomiting, changes in bowel or urinary habits, fever, or other constitutional symptoms. There was no previous history of similar pain, abdominal trauma, or recent heavy exertion.

On examination, the patient was alert, oriented, and hemodynamically stable. Vital signs were within normal limits, and he was afebrile. Abdominal examination revealed localized tenderness and mild guarding over the epigastric and RUQ regions, with a palpable soft, well-circumscribed swelling measuring approximately 3 × 4 cm. There was no distension, rebound tenderness, or other peritoneal signs. The remainder of the systemic examination was unremarkable.

**Figure 1:**
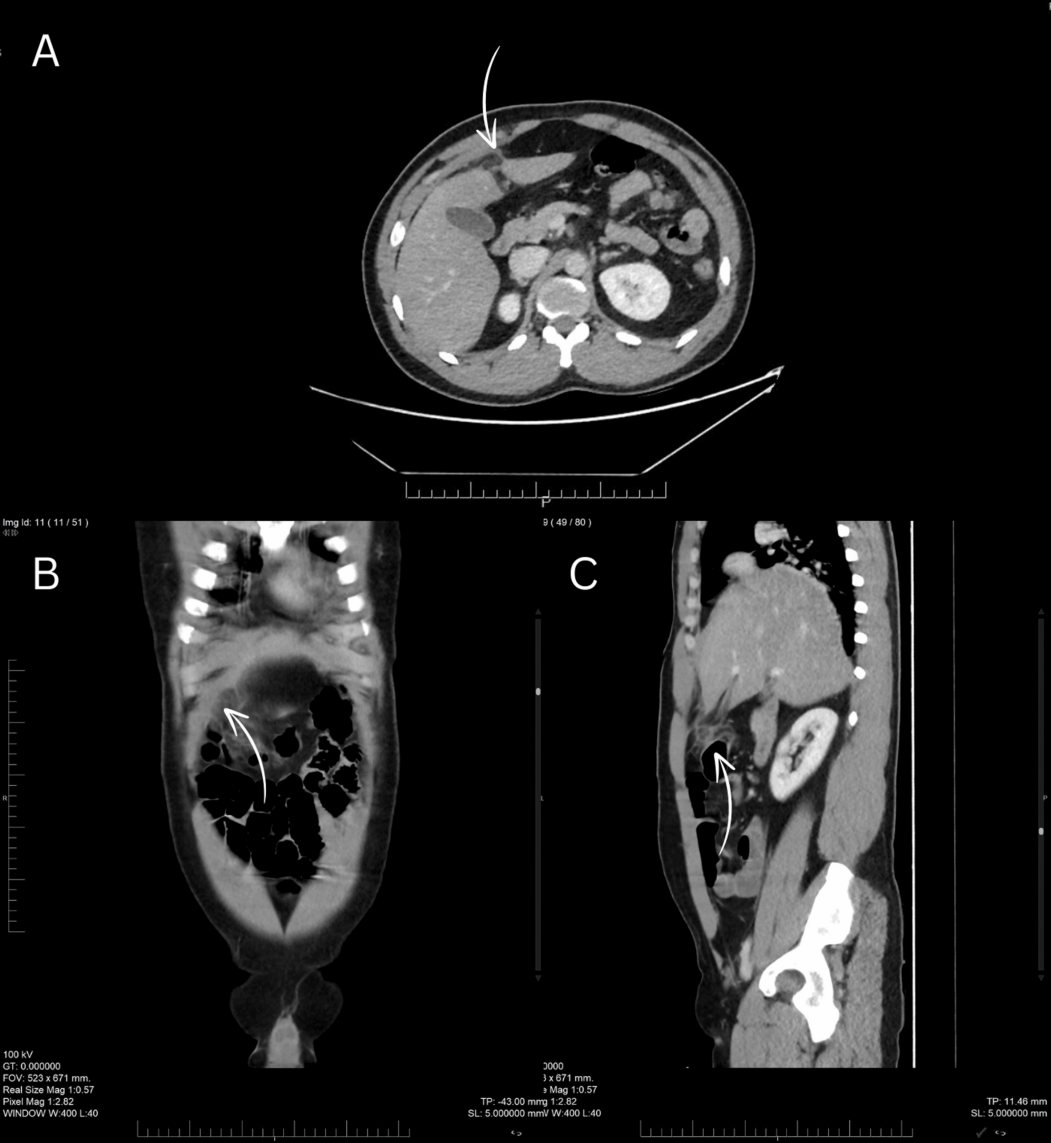
Contrast-enhanced CT scan of the abdomen showing features suggestive of falciform ligament appendagitis. (A) Axial, (B) coronal, and (C) sagittal views demonstrate a fat-attenuated oval lesion with surrounding inflammatory changes adjacent to the falciform ligament (arrows).

Initial laboratory investigations showed a white blood cell count of 10.24 × 10^9^/L, hemoglobin 16.8 g/dL, and platelet count of 295 × 10^9^/L. Liver enzymes were within normal limits, except for a slightly elevated alanine aminotransferase (ALT) of 60 U/L. Other biochemical parameters were unremarkable. An ultrasound of the abdomen performed prior to admission suggested a possible rectus sheath hematoma; however, to further delineate the lesion, a contrast-enhanced CT scan of the abdomen was obtained. CT imaging demonstrated a fat-density, oval-shaped lesion adjacent to the falciform ligament with surrounding inflammatory stranding, consistent with falciform ligament appendagitis (Figure 1). No rectus sheath hematoma, gallbladder pathology, or intra-abdominal collection was identified.

The patient was admitted under the General Surgery department and managed conservatively. He was initially kept nil per os (NPO) and started on intravenous antibiotics, analgesia including paracetamol 1g every six hours and naproxen 250mg twice daily, and intravenous fluids. Daily laboratory monitoring and clinical assessments were performed.

On hospital day 2, the patient continued to report localized RUQ pain but remained afebrile and hemodynamically stable. Repeat physical examination showed persistent but improving tenderness without guarding or distension. Laboratory investigations were repeated, with results showing stable hemoglobin and platelet counts.

By hospital day 3, the patient’s condition had notably improved. He was pain-free at rest, tolerated clear fluids, and exhibited no new symptoms. Abdominal examination revealed only mild epigastric tenderness with no peritoneal signs. The patient was started on a regular diet, and his intravenous fluids were gradually reduced.

On hospital day 4, the patient was asymptomatic, and physical examination was completely normal. Repeat laboratory results showed a normalized WBC count of 7.8 × 10^9^/L, while hemoglobin (15.2 g/dL) and platelets (266 × 10^9^/L) remained within normal limits (Table 1). The patient was deemed clinically stable for discharge.

**Table 1:** Summary of laboratory values.

Parameter	Reference range	Day 1	Day 4
Complete blood count			
Hemoglobin (g/dL)	13.2-16.6	16.8	15.2
Total red blood cell count (x10^12^/L)	4.35-5.65	5.94	5.48
Total leucocyte count (x10^9^/L)	3.4-9.6	10.24	7.80
Platelet count (x10^9^/L)	150-400	295	266
Biochemistry			
Serum creatinine (µmol/L)	65-104	78	79
Serum urea (mmol/L)	3.2-8.2	4.4	3.8
Bilirubin (total) (µmol/L)	5-21	19	17
Alkaline phosphatase (U/L)	56-116	77	73
Electrolytes			
Sodium (mmol/L)	136-145	138	138
Potassium (mmol/L)	3.4-4.5	4.6	4
Liver function test			
Protein (total) (g/L)	57-82	71	65
Albumin (g/L)	35-52	45	42

The patient showed complete clinical improvement during hospitalization and was discharged in stable condition. At the time of discharge, he was asymptomatic, tolerating oral intake, and did not require ongoing analgesia. No scheduled outpatient follow-up was arranged, and the patient was advised to return if symptoms recurred.

## Discussion

The falciform ligament is a sagittal double fold of the peritoneum. It extends from the liver to the anterior abdominal wall. The left inferior phrenic and middle hepatic arteries provide the falciform ligament's primary blood supply. The falciform ligament serves as an excellent material for covering organ defects and repairing damaged surfaces within the abdominal cavity due to its extensive size, adequate length, rich blood supply, and well-developed vascular connections [3].

Fatty-falciform ligament appendage torsion (F-FLAT) is a rare condition caused by torsion of the extraperitoneal lipomatous appendage of the falciform ligament, leading to ischemia and aseptic fat necrosis. This results in falciform ligament appendagitis, an entity that clinically mimics other intraperitoneal focal fat infarctions (IFFIs) such as omental infarction and epiploic appendagitis, often making diagnosis challenging [2,4].

To date, only seven cases of falciform ligament appendagitis have been explicitly reported, highlighting the rarity of this condition. Within the spectrum of IFFI of the falciform ligament, few reports have specifically used the term "falci­form ligament appendagitis" or "hepatic falciform ligament appendagitis." A focused review of peer-reviewed case reports identified seven previously reported patients meeting this terminology (Table 2). Our case, therefore, represents the eighth reported patient diagnosed with falciform ligament appendagitis using this specific label. Additional cases with similar pathology have been published under alternative terms, such as "torsion of a fatty appendage of the falciform ligament," "falciform ligament fat infarction," or "fat necrosis/inflammation of the falciform ligament"; however, these were not included in this count, as they do not explicitly mention appendagitis in the title or abstract.

**Table 2:** Reported cases of falciform ligament appendagitis diagnosed using imaging modalities. CT: computed tomography; MRI: magnetic resonance imaging; MRCP: magnetic resonance cholangiopancreatography

Author(s)	Number of cases	Age group	Year	Imaging modality	Management
Rousslang et al. [5]	1	Adult	2020	Ultrasound, CT	Conservative
Miura et al. [6]	2	Adult	2022	Ultrasound, CT; Ultrasound, MRI	Conservative
Muleta et al. [7]	1	Pediatric	2024	Ultrasound	Conservative
Chen et al. [8]	2	Adult	2024	Ultrasound, CT, MRCP; CT	Conservative
Manso and Agostinho [2]	1	Adult	2024	CT	Conservative

Falciform ligament appendagitis occurs slightly more often in males than in females, with an almost equal sex ratio (approximately 1:1) and a median age of around 59.5 years. The only reported risk factor is obesity, particularly an increase in abdominal visceral fat, although available data remain limited [9]. Other falciform ligament pathologies include internal herniation through congenital defects, as well as congenital, infectious, or neoplastic cysts and lipomas. In some cases, isolated necrosis of the falciform ligament may occur without any associated abnormalities [10].

Clinically, patients typically present with RUQ or epigastric pain that is usually non-radiating, accompanied by low-grade fever and leukocytosis [5,9]. Ultrasound can help locate and identify the lesion through real-time dynamic assessment with the patient. Using a high-frequency probe may further enhance diagnostic accuracy when FLA is suspected; however, it is insufficient for distinguishing between different possible causes [6,11].

CT imaging is crucial for accurately diagnosing gastroduodenal and pancreatic conditions, as well as rare mimicking entities such as IFFI [11]. It typically demonstrates falciform ligament appendagitis as a focal, well-defined area of fat attenuation adjacent to the falciform ligament, surrounded by a thin hyperattenuating rim, with or without a central dot representing a thrombosed draining vein [6]. MRI may also detect the lesion, typically appearing as a fat-signal area with surrounding inflammation, but its role is secondary compared to CT [12].

Falciform ligament appendagitis is a self-limiting condition that typically resolves within three to 14 days with conservative management, without the need for surgical intervention. Very rare complications may include adhesion formation, peritonitis, abscess development, or calcified peritoneal loose bodies [1,10]. A few reported cases underwent surgery, mainly because of limited awareness of the condition and its typically self-limiting course. Another indication for laparotomy was the concern for necrosis and gangrene, as documented in some reports [13,14].

## Conclusions

Falciform ligament appendagitis is a rare but self-limiting cause of acute RUQ pain that can be accurately diagnosed with CT imaging, allowing safe conservative management and avoiding unnecessary surgical approaches.
